# Impact of the WHO emergency care toolkit on mortality in Zambia: an implementation-effectiveness hybrid study

**DOI:** 10.1136/bmjgh-2025-019729

**Published:** 2025-11-09

**Authors:** Morgan C Broccoli, James Nonde, Kephas E Mwanza, Julia Dixon, Jennifer Pigoga Hart, Lauren Lai King, Patricia Chibesakunda, Natasha Chenga, Winnie Kunda, Azmach Hadush, Godfrey Phiri, Alex Makupe, Lee A Wallis, Mwiche Chiluba

**Affiliations:** 1 Department of Emergency Medicine, Brigham and Women’s Hospital, Boston, Massachusetts, USA; 2 Ndola Teaching Hospital, Ndola, Copperbelt Province, Zambia; 3 Solwezi General Hospital, Solwezi, North-Western Province, Zambia; 4 Department of Emergency Medicine, University of Colorado School of Medicine, Aurora, Colorado, USA; 5 African Federation for Emergency Medicine, Bellville, South Africa; 6 Division of Emergency Medicine, University of Cape Town Faculty of Health Sciences, Cape Town, South Africa; 7 University Teaching Hospital, Lusaka, Lusaka Province, Zambia; 8 WHO Country Office for Zambia, Lusaka, Lusaka Province, Zambia; 9 Mobile and Emergency Health Services, Zambia Ministry of Health, Lusaka, Lusaka Province, Zambia; 10 Clinical Care and Diagnostic Services, Zambia Ministry of Health, Lusaka, Lusaka Province, Zambia; 11 Clinical Services and Systems, Integrated Health Services, WHO, Geneva, Switzerland

**Keywords:** Africa South of the Sahara, Delivery of Health Care, Global Health, Health systems, Universal Health Care

## Abstract

**Introduction:**

Low‐ and middle-income countries (LMICs) suffer the highest rates of death and disability due to emergency conditions. Strong emergency care systems reduce the morbidity, acquired disability and mortality associated with a variety of emergency conditions and have the potential to impact over half of the annual deaths in LMICs. The WHO emergency care toolkit (ECT) is an open access bundle of simple interventions to support care of the acutely ill and injured in LMICs. The aim of this study was to assess the impact of the WHO ECT on facility-based mortality at eight provincial-level hospitals in Zambia.

**Methods:**

This study used an implementation-effectiveness hybrid design. Prospective data on mortality from a set of sentinel emergency conditions (paediatric diarrhoea, pneumonia, asthma, diabetic ketoacidosis (DKA), postpartum haemorrhage and injury) were collected for 8 months pre-implementation and post-implementation at eight provincial-level hospitals in Zambia. Case fatality ratios (CFRs) were calculated across intervention periods, and Z scores identified significant changes. Logistic regression analyses were conducted to identify associations between independent variables and mortality.

**Results:**

A total of 7333 patients presented to the eight study sites with sentinel conditions: 4105 patients before and 3228 following implementation of the WHO ECT. Across all sentinel conditions, a significant decrease of 33.6% was seen in the crude CFR, and the odds of mortality were 35.7% lower after adjusting for confounders. Paediatric diarrhoea (−60.1%, p=0.048), DKA (−59.2%, p=0.001) and pneumonia (−48.1%, p<0.001) saw significant decreases in CFRs following WHO ECT implementation, and downward trends were seen for asthma and postpartum haemorrhage.

**Conclusion:**

Implementation of the WHO ECT in eight Zambian hospitals showed a 35.7% reduction in adjusted odds of mortality from six sentinel conditions. The WHO ECT is an effective means of strengthening emergency care and improving patient outcomes in LMICs.

**WHAT IS ALREADY KNOWN ON THIS TOPIC**

- Strong emergency care systems are needed to address the excess disability and death from emergency conditions in low‐ and middle-income countries (LMICs).
- The WHO has created the WHO emergency care toolkit (ECT) to assist countries with strengthening their emergency care systems.
- Although the WHO ECT has been implemented in multiple countries, there is limited evidence of its impact on patient-centred outcomes such as mortality.

**WHAT THIS STUDY ADDS**

- To our knowledge, this study is the first to provide evidence of a mortality benefit associated with the implementation of the WHO ECT in low-resource settings. After analysing data from eight provincial-level hospitals in Zambia, we found a significant 35.7% reduction in adjusted odds of mortality from six sentinel conditions following the introduction of the WHO ECT.
- Given the importance of emergency care for reducing morbidity and mortality in LMICs, and the emphasis WHO has placed on strengthening emergency care systems globally, our results provide evidence that implementing basic emergency care training, triage and designation of a resuscitation area can effectively reduce emergency-related mortality in LMICs.

**HOW THIS STUDY MIGHT AFFECT RESEARCH, PRACTICE OR POLICY**

- The findings of this study highlight the critical role of emergency care system strengthening in reducing preventable deaths in LMICs. The WHO ECT offers an open access, scalable package of interventions that can improve emergency care outcomes when effectively implemented.
- Policymakers and health system leaders should consider integrating the WHO ECT into national emergency care system strengthening strategies to reduce morbidity and mortality.
- Further research is needed to explore long-term sustainability, cost-effectiveness and the feasibility of broader implementation across diverse settings.

## Introduction

 Low- and middle-income countries (LMICs) suffer the highest rates of death and disability due to emergency conditions resulting from trauma, obstetric complications and acute complications of both communicable and non-communicable diseases.^1^ This is due both to the higher burden of emergency conditions and vast discrepancies in outcomes for these conditions in LMICs.^2 3^ Unfortunately, many of these emergency conditions disproportionately affect the young and are associated with substantial societal and economic costs.^3^,^5^

Strong emergency care systems (ECS) have been shown to reduce the morbidity and mortality associated with a variety of emergency conditions and have the potential to ameliorate over half of the annual deaths in LMICs.^2 6 7^ Simple individual interventions aimed at strengthening emergency care provision in LMICs through the implementation of formal triage, dedicated resuscitation areas, short courses and clinical protocols have shown significant reductions in mortality.^28^,^10^

Emergency care not only has the potential to address a large burden of disease in LMICs, but it is also the way in which a large proportion of the population accesses the healthcare system.^2^ In recognition of the critical importance of ECS for ensuring universal health coverage, the World Health Assembly (WHA) has adopted several resolutions on emergency care, most recently with resolution WHA 76.2, which calls for integrated emergency, critical and operative care services to ensure universal health coverage and protect against health emergencies.^11^ The WHO has created multiple tools to assist countries with strengthening their emergency care systems and ensuring universal access.^12^

Five of these interventions are bundled in the WHO emergency care toolkit (ECT), a set of prioritised, open access resources designed to be implemented in emergency units in resource-limited settings.^13^ The five components of the WHO ECT are: (1) the Basic Emergency Care (BEC) course, an open access clinical training course for frontline healthcare providers managing acute illness and injury; (2) guidance on resuscitation area designation, which provides a standardised approach to staffing, equipping and organising resuscitation areas to ensure that the delivery of emergency care to the sickest patients is optimised; (3) emergency care checklists, which ensure a systematic approach to providing key life-saving elements of emergency medical and trauma care; (4) a triage training package using the Interagency Integrated Triage Tool (IITT) and (5) standardised clinical forms for emergency unit documentation.^13^

In the last decade, Zambian stakeholders, including the Ministry of Health (MoH), have recognised the need to improve emergency care services in the country. The MoH has conducted multiple needs assessments for emergency care, including the WHO’s Emergency Care System Assessment, and identified strengthening hospital emergency units through triage, education and accreditation as a priority (Ministry of Health Zambia, National Stakeholders Consultative Meeting on Emergency Health Services: Action Plan). Although there are currently no formal training programmes for emergency medicine specialists in Zambia, there is an emergency and trauma nursing programme as well as short courses for healthcare providers and community members in basic emergency care.^14^,^16^ The MoH also sponsored physicians to complete specialist training in South Africa, who have returned to Zambia to lead emergency care development efforts.^17^

As part of their emergency care strengthening activities, the Zambian MoH collaborated with WHO to implement the WHO ECT in eight hospitals throughout Zambia with the goal of improving patient outcomes for emergency conditions. Although the WHO ECT has been implemented in over 20 countries across multiple resource settings, there is limited evidence of its impact on mortality. The preliminary data analysis of the WHO ECT pilot in Uganda and Tanzania showed a promising composite 48-hour mortality decrease of 45% across the selected sentinel conditions, but these data are still unpublished.^18^ The aim of this study was to assess the impact of the WHO ECT on facility-based mortality in Zambia.

## Methods

**Figure 1:**
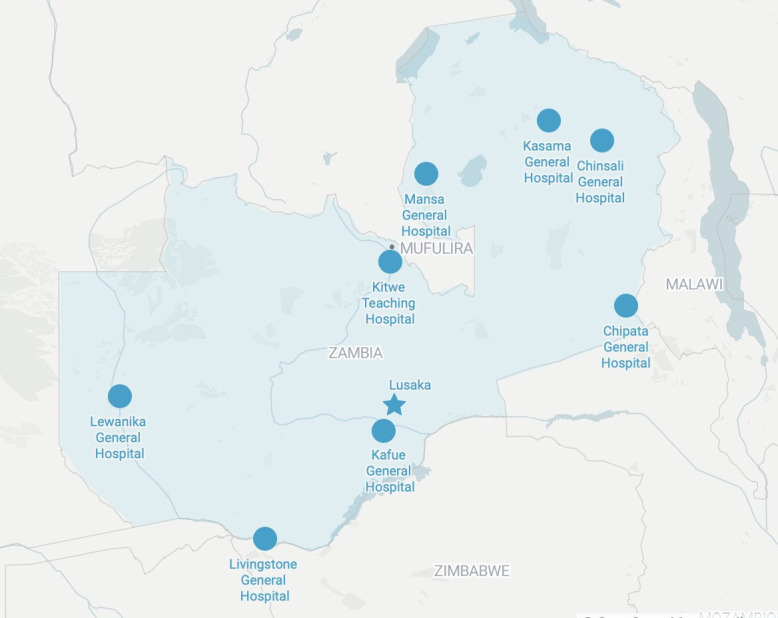
Locations of included facilities (n=8) throughout Zambia.

This study used a quasi-experimental, type 2 implementation-effectiveness hybrid design.^19^ Prospective data on mortality from a set of six sentinel emergency conditions (paediatric diarrhoea, pneumonia, asthma, diabetic ketoacidosis (DKA), postpartum haemorrhage and injury) were collected pre-implementation and post-implementation at eight hospitals in Zambia (figure 1). These six sentinel conditions were chosen as they are considered to be highly amenable to timely, quality emergency care.^20^

### Hospital selection

Provincial hospitals were selected for this study to ensure they would receive sufficient volumes of the sentinel emergency conditions during the study period. Provincial hospitals in Zambia serve a catchment area of 200 000–800 000 people and provide specialist services including internal medicine, general surgery, paediatrics, obstetrics and gynaecology, and intensive care.^21^ To mitigate confounding, hospitals were excluded if they had an emergency medicine specialist working at the hospital or had already received substantial training in emergency care. The intervention was conducted in 8 of the 10 provinces in Zambia. All included hospitals are managed by the Zambian Ministry of Health.

### Intervention

Prior to implementation, the MoH and the Emergency Medicine Society of Zambia reviewed the WHO ECT and customised the implementation package to fit the local context. The MoH and the Emergency Medicine Society of Zambia chose to implement three components of the WHO ECT without modification: the BEC course, guidance on resuscitation area designation and emergency care checklists. For triage, stakeholders opted to substitute the South African Triage Scale (SATS) for the IITT, as SATS is already widely used in Zambian tertiary hospitals and use of a common triage tool was felt to be key to successful implementation. The final component of the ECT, standardised clinical forms for emergency unit documentation, was not implemented.

Two champions were selected from each facility to lead implementation of the Zambian modified WHO ECT, hereafter referred to as the ECT, at their hospital together with the Emergency Medicine Society of Zambia to ensure sustainability of the intervention. The implementation process will be described in more detail in a separate publication.

### Data collection

Each site collected clinical data before and after the ECT implementation period. Power calculations were used to determine the minimum sample size required to detect a 45% reduction in mortality for aggregated sentinel conditions following ECT implementation, based on mortality reduction findings from original pilot studies conducted in a similar low-income country, necessitating a minimum of 1500 patients each in the pre-intervention and post-intervention periods. Based on previously determined patient presentation volumes for the sentinel conditions of interest, it was estimated that 8-month data collection periods would result in sufficient sample sizes across the eight participating facilities. A 4-week washout period was implemented on both sides of the intervention. The primary outcome of interest was overall facility-based mortality (defined as mortality at any point during a patient’s emergency unit and/or inpatient facility stay) from sentinel conditions. Data related to patient demographics, clinical characteristics and interventions, facility disposition and other process indicators were collected internally on a secure District Health Information Software 2 (DHIS-2) instance during the study period. (Trained data collectors followed an established protocol to identify eligible patients pre-ECT intervention and post-ECT intervention and extract elements of interest from routinely captured hospital charts into the DHIS-2 platform.

### Data analysis

We assessed descriptive statistics in the pre-intervention and postintervention cohorts and by age, condition and facility. Case fatality ratios (CFRs), which define the total number of deaths as a proportion of the total number of reported cases of a sentinel condition during a time period, and Z scores were used to identify statistically significant changes in these unadjusted ratios. Bivariate analyses were conducted to identify potential associations between independent variables and mortality using t-tests and χ^2^ tests for continuous and categorical variables, respectively. We then performed multivariable logistic regression to compare the risk of facility-based mortality before and after the intervention. All covariates were included in the final model based on clinical relevance and prior knowledge. A two-tailed p-value of <0.05 was interpreted as statistically significant. All statistical analyses were conducted using SAS OnDemand for Academics (SAS Institute, Cary, North Carolina, USA).

## Results

A total of 7333 patients presenting to the eight participating study sites with sentinel conditions were included in this study: 4105 patients prior to and 3228 following implementation of the ECT (table 1). Individuals missing the primary outcome of interest and/or covariates (n=81, 1.1%) were excluded from this analysis.

**Table 1:** Demographic and clinical characteristics of included patients before (n=4105) and after (n=3228) implementation of the Zambian modified WHO emergency care toolkit

Characteristic	Overall (n=7333)	Pre-implementation (n=4105)	Post-implementation (n=3228)
Demographics			
Age (median (IQR))	35 (14.9–51.7)	37.4 (29–52.4)	20 (8.3–50.9)
Sex (female)	3114 (42.5)	1636 (39.9)	1478 (45.8)
Sentinel condition			
Asthma	758 (10.3)	406 (9.9)	352 (10.9)
Diabetic ketoacidosis	451 (6.2)	199 (4.8)	252 (7.8)
Paediatric diarrhoea	348 (4.7)	182 (4.4)	166 (5.1)
Injury	3919 (53.4)	2292 (55.8)	1627 (50.4)
Pneumonia	1652 (22.5)	891 (21.7)	761 (23.6)
Postpartum haemorrhage	205 (2.8)	135 (3.3)	70 (2.2)
Facility disposition			
Discharged home	6619 (90.3)	3683 (89.7)	2936 (91)
Left before completing care	108 (1.5)	44 (1.1)	64 (2)
Transferred to another facility	166 (2.3)	89 (2.2)	77 (2.4)
Death	440 (6)	289 (7)	151 (4.7)

Individuals presenting during the post-implementation period were younger (median 20 years; IQR 8.3–50.9 years) and more frequently female (45.8%) compared with those in the pre-implementation cohort (median 37.4 (IQR 29–52.4) years of age; 39.9% female). Injury accounted for the majority of patients presenting with sentinel conditions, including 53.4% (n=2292) prior to implementation and 55.8% (n=1627) following the intervention. Pneumonia was also common, seen in 21.7% (n=891) and 23.6% (n=761) of pre-implementation and post-implementation patients, respectively. Asthma and DKA comprised 9.9% (n=406) and 4.8% (n=199) of patients prior to the intervention and 10.9% (n=352) and 7.8% (n=252) following. Rates of presentation with diarrhoea in children under 5 years of age were similar in the pre-intervention (4.4%, n=182) and post-intervention (5.1%, n=166) periods. Only 3.3% (n=135) of pre-intervention patients presented with postpartum haemorrhage and 2.2% (n=70) post-intervention.

Nearly all individuals (89.7% pre-implementation and 91% post-implementation) were eventually discharged home from the hospital to which they initially presented. A total of 289 patients (7%) with sentinel conditions died at facilities prior to the intervention, and 151 (4.7%) after implementation. Few patients left before completing care (1.1% before and 2% after implementation) or were transferred to different facilities (2.2% before and 2.4% after implementation).

Most patients in the pre-intervention period were between the ages of 18 and 59 years (n=2738, 66.7%), with few below 5 years of age (n=270, 6.6%), between 5 and 18 years (n=408, 9.9%), and above 60 years (n=689, 16.8%). Ages were more evenly distributed in the post-intervention period: approximately one-third (n=1218, 37.7%) were 18–59 years of age, 29.4% (n=949) were 5–18 years, 16.8% were 60 years or older, and 16% (n=518) were under 5 years of age.

Additional information on demographics and facility dispositions for individual sentinel conditions before and after ECT implementation can be found in online supplemental Table 1).

**Figure 2:**
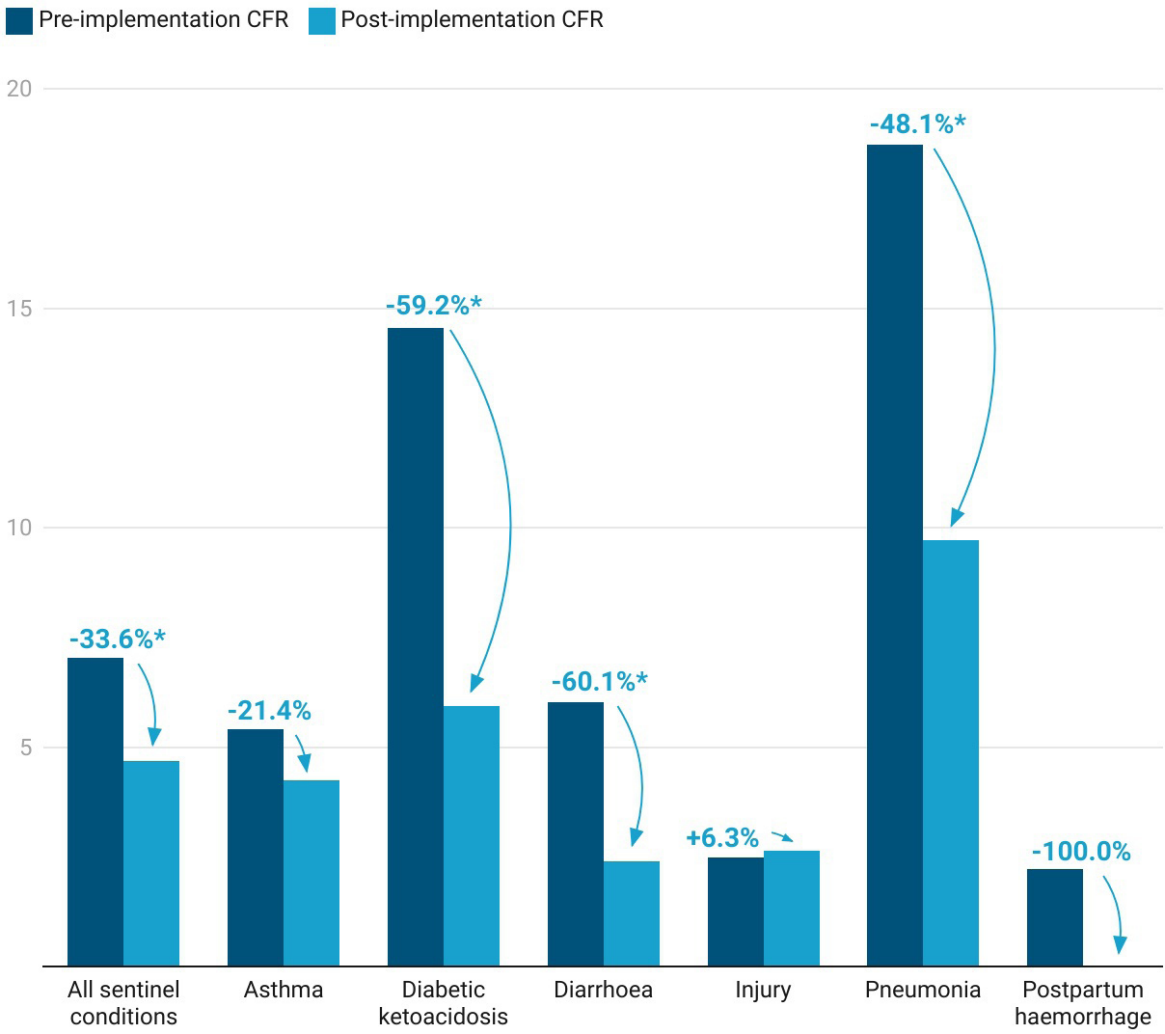
Changes in CFRs in individuals presenting with sentinel conditions, pre-ECT and post-ECT implementation in Zambia. CFRs, case fatality ratios; ECT, emergency care toolkit.

Across all sentinel conditions, a significant decrease of 33.6% was seen in the crude CFR (table 2). Three conditions, paediatric diarrhoea (−60.1%, p=0.048), DKA (−59.2%, p=0.001) and pneumonia (−48.1%, p<0.001), also saw significant decreases in CFRs (figure 2). Although not statistically significant, a downward trend was seen in the asthma-related CFR post-intervention, which decreased by 21.4% (p=0.230). Postpartum haemorrhage also saw a non-significant decrease (−100%), though the number of fatalities prior to the intervention was already very low (n=3, CFR: 2.2%). Only one condition, injury, saw a non-significant increase in CFR, from 2.5% to 2.6%.

**Table 2:** Cases and associated fatalities in included patients before (n=4105) and after (n=3228) implementation

	Pre-implementation			Post-implementation			% Change in CFR	P value
	Cases (n)	Fatalities (n)	CFR (%)	Cases (n)	Fatalities (n)	CFR (%)		
Overall (all sites and conditions)	4105	289	7	3228	151	4.7	−33.6	<0.001*
Age								
<5 years	270	18	6.7	518	16	3.1	−53.7	0.010*
5 to <18 years	408	16	3.9	949	45	4.7	20.9	0.252
18 to <60 years	2738	170	6.2	1218	49	4	−35.2	0.003*
≥60 years	689	85	12.3	543	41	7.6	−38.8	0.003*
Sentinel conditions								
Asthma	406	22	5.4	352	15	4.3	−21.4	0.230
Diabetic ketoacidosis	199	29	14.6	252	15	6	−59.2	0.001*
Paediatric diarrhoea	182	11	6	166	4	2.4	−60.1	0.048*
Injury	2292	57	2.5	1627	43	2.6	6.3	0.380
Pneumonia	891	167	18.7	761	74	9.7	−48.1	<0.001*
Postpartum haemorrhage	135	3	2.2	70	0	0	−100	0.091
Facilities								
Facility 1	506	18	3.6	426	28	6.6	83.3	0.017*
Facility 2	459	34	17.4	308	9	2.9	−83.3	0.004*
Facility 3	913	105	11.5	510	39	7.6	−33.9	0.010*
Facility 4	508	24	4.7	419	11	2.6	−44.7	0.048*
Facility 5	122	9	7.4	71	4	5.6	−24.3	0.321
Facility 6	733	50	6.8	788	34	4.3	−36.8	0.016*
Facility 7	317	21	6.6	198	6	3	−54.5	0.038*
Facility 8	547	28	5.1	508	20	3.9	−23.5	0.179

* Statistical significance at a p value <0.05.

CFR, case fatality ratio.

Significant decreases in CFR were seen following ECT intervention in three of the four age groups included in this study: under 5 years of age (−53.7%, p=0.010), between 18 and 59 years of age (−35.2%, p=0.003) and 60 years and above (−38.8%, p=0.003) (table 2). The CFR for those 5–18 years of age rose from 3.9% to 4.7% (p=0.252).

Several covariates were identified as predictive of survival in our bivariate analysis: time period (pre-intervention or post-intervention, p<0.001), age (continuous, p<0.001), sentinel condition (p<0.001) and facility (p<0.001). While a sensitivity analysis identified that sex did not affect estimates for other variables, it was included in the final model due to its clinical relevance. In an adjusted logistic regression of survival, mortality was 35.7% (95% CI 55.5% to 85.3%) less likely in those presenting in the post-ECT implementation period versus the pre-ECT implementation period (table 3).

**Table 3:** Logistic regression of factors influencing survival in individuals presenting to eight Zambian hospitals

Characteristic		Unadjusted OR (95% CI)	Adjusted OR (95% CI)
Time period (reference=pre-implementation period)	Post-implementation period	0.65 (0.53 to 0.79)	0.64 (0.51 to 0.81)
Age (categorical) (reference <5 years)	5 to <18 years	1.30 (0.90 to 1.88)	1.36 (0.82 to 2.52)
	18 to <60 years	1.04 (0.68 to 1.60)	1.45 (0.84 to 2.49)
	≥60 years	2.53 (1.71 to 3.73)	2.27 (1.35 to 3.82)
Sex (reference=male)	Female	1.14 (0.94 to 1.38)	0.96 (0.78 to 1.17)
Facility (reference=Facility 1)	Facility 2	0.53 (0.37 to 0.75)	0.49 (0.34 to 0.71)
	Facility 3	0.46 (0.33 to 0.65)	0.37 (0.26 to 0.53)
	Facility 4	0.35 (0.24 to 0.51)	0.38 (0.26 to 0.56)
	Facility 5	0.64 (0.36 to 1.16)	0.42 (0.23 to 0.77)
	Facility 6	0.52 (0.39 to 0.69)	0.64 (0.48 to 0.86)
	Facility 7	0.49 (0.32 to 0.75)	0.44 (0.28 to 0.68)
	Facility 8	0.42 (0.30 to 0.59)	0.43 (0.30 to 0.61)
Sentinel condition (reference=postpartum haemorrhage)	Diabetic ketoacidosis	7.28 (2.23 to 23.73)	6.98 (2.11 to 23.14)
	Asthma	3.46 (1.05 to 11.32)	3.29 (0.99 to 10.93)
	Paediatric diarrhoea	3.03 (0.87 to 10.61)	4.96 (1.28 to 19.19)
	Injury	1.76 (0.55 to 5.61)	1.70 (0.53 to 5.47)
	Pneumonia	11.5 (3.65 to 36.25)	11.60 (3.63 to 37)

## Discussion

This study found a 33.6% crude reduction in case fatalities across six sentinel conditions after implementation of the Zambian modified WHO ECT at eight provincial-level hospitals in Zambia. When adjusting for confounders, the odds of mortality were 35.7% lower in the post-intervention period than in the pre-intervention period and remained statistically significant. This reduction in mortality persisted across multiple clinical conditions and age groups. Three sentinel conditions had statistically significant reductions in mortality: diarrhoea in children under 5 years of age, pneumonia and DKA.

The results of this study are in line with the few others that have attempted to measure the impact of a basic, systematic approach to emergency care on patient-centred outcomes in LMICs.^8 9 18^ Possible explanations for this reduction in mortality include implementation of appropriate triage, reduced time to being seen by a doctor or senior clinician and reduced time to critical interventions.^22^ While time to intervention was not assessed in this study, timely recognition and intervention for emergency conditions is a cornerstone of the WHO BEC training.^13^

Three sentinel conditions had statistically significant reductions in mortality: diarrhoea in children under 5 years of age, pneumonia and DKA. It is not surprising that these conditions benefit from a systematic approach to emergency care, as their treatments are concrete, simple, time sensitive and highly impactful. For example, in children with acute diarrhoea, oral rehydration solution for dehydration significantly reduces mortality, is low cost and is easily administered.^23^ A cornerstone of DKA management is intravenous fluids, and DKA protocols in low-resource settings have led to reduced mortality.^24 25^ Although it was not statistically significant, there was also a downward trend in asthma-related case fatalities post-intervention. Asthma is another acute medical condition which is treated with simple, timely interventions, including early recognition through triage and a structured approach to assessment and treatment as taught in BEC.^2^

Interestingly, there was no statistically significant change in mortality from injury during the post-intervention period. This may be because ‘injury’ is a less specific condition than pneumonia or asthma and, in fact, encompasses a wide range of patient presentations (eg, traumatic brain injury, chest trauma and intra-abdominal injury) which may have very different acuity. Injury outcomes are also affected by many more points in the healthcare system, including availability and type of imaging modality, availability of blood transfusion, surgical capacity and expertise, and intensive care. Other studies, however, have shown a mortality reduction in injured patients after implementation of standardised trauma protocols or focused trauma education.^9 10 26^

There was a 100% reduction in mortality from postpartum haemorrhage in the post-intervention period; this was not statistically significant, as the absolute number of fatalities was low even in the pre-intervention period. This may be due to the longstanding emphasis on reducing maternal mortality in Zambia.^27^ Maternal health has been a global priority since the Millennium Development Goals were signed in 2000, and thanks to dedicated programming and funding, Zambia decreased its maternal mortality ratio by more than half in subsequent decades.^27^

Three of the four age groups also saw a significant reduction in mortality postintervention, with over a 50% reduction in CFR for children under 5 years of age. Globally, diarrhoea remains one of the leading causes of death in this group.^28^ Implementation of the WHO ECT could have a significant impact on outcomes for this population. While this decrease in CFR for children under 5 years of age was significant, shifts in the distribution of sentinel conditions pre-intervention and post-intervention may also be contributing to changes in CFR. For children under 5 years of age, the post-implementation cohort was much larger than the pre-implementation; therefore, the median age of patients was substantially younger in our post-implementation cohort. This was driven primarily by an increase in children with injury, the outcomes of which tend to be worse in children, particularly in regions without dedicated paediatric trauma centres like Zambia.^29 30^ While conditions were adjusted for in our cohort-wide regression, they were not in the age-specific analyses. The impact of a greater injury burden in the under-five group, which evidence suggests would increase mortality, could lead to a small underestimate of ECT impact in this subcohort.

It is also notable that those over age 60 also saw a 38.8% reduction in mortality. Population projections indicate the vast majority of those over 60 will be living in LMICs by 2050.^31^ Concurrently, emergency units will inevitably see more myocardial infarctions, strokes, injuries and pneumonia, among other clinical conditions that require timely interventions.^2 32^ The only age group that did not see a reduction in mortality was children aged 5–18 years. One possible explanation is the large proportion of injured patients aged 5–18 years. In this study, the heterogeneous nature of the injury cohort and limited details regarding injury mechanism and severity limit interpretation.

Although our study did not assess ECT impacts on specific sexes, there was an increase in the proportion of females in the post-implementation cohort. The influence of sex on mortality risk is varied across the sentinel conditions studied. Injury mortality risk is particularly sensitive across sexes; males tend to present with more severe violence-related injuries and generally experience higher mortality rates from injuries across all age groups.^30 33^ Mortality from respiratory conditions, such as pneumonia, varies by age, with younger females and older males being at greater risk of death.^34^ Although we did not conduct a sex-specific assessment of sentinel conditions on mortality, our bivariate analyses detected no significant differences in mortality likelihood across males and females.

This study had several limitations. The study was powered to detect an overall mortality reduction in the combined six sentinel conditions; it was not powered for any single sentinel condition or age group. Future studies should enrol more patients and be powered to measure outcomes related to individual conditions of interest. The study initially planned to assess mortality at 48 hours from presentation, but many of the patient forms were incomplete and 60.5% of facility dispositions were missing a timestamp. We were therefore only able to assess overall mortality during hospitalisation. And since this study assessed overall mortality, there are many other factors which can influence this outcome, which we were not able to measure. Finally, there were challenges with incomplete data capture that affected other data points, including triage score, vital signs and provision of key interventions. Due to this incomplete data capture, we were unable to assess the impact of the WHO ECT on process measures such as triage rate, vital sign capture and provision of key interventions. One factor may have been trying to collect too many data points on enrolled patients, reflecting the need for improved data collection systems in the future. Although WHO funded the study and contributed to its design and reporting, they were not involved in data collection, analysis or interpretation.

## Conclusion

LMICs have a high burden of emergency conditions leading to excess disability and death, particularly in vulnerable populations. Implementation of the WHO ECT in eight provincial-level hospitals in Zambia reduced the odds of mortality from six sentinel conditions by 35.7%. This study supports the growing body of evidence that strengthening emergency care provision in LMICs can address a large burden of disease and decrease excess disability and death.

## Supplementary material

10.1136/bmjgh-2025-019729 online supplemental file 1

## Data Availability

Data may be obtained from a third party and are not publicly available.
