# Supplementary material for: Impact of the WHO emergency care toolkit on mortality in Zambia: an implementation-effectiveness hybrid study
Source: BMJ Glob Health. 2025 Nov 9;10(11):e019729. doi: 10.1136/bmjgh-2025-019729 (PMC12598988; doi:10.1136/bmjgh-2025-019729)
Supplement: online supplemental file 1 [file bmjgh-10-11-s001.pdf]

Supplemental Table 1. Demographic and disposition characteristics of included patients before (n=4,105) and after (n=3,228) implementation, by core condition.

**Implementation period**	**Pre-implementation** (n=4,105)						**Post-implementation** (n=3,228)					
**Key condition**	**Asthma** (n=406)	**Diabetic ketoacidosis** (n=199)	**Paediatric diarrhoea** (n=182)	**Injury** (n=2292)	**Pneumonia** (n=891)	**Postpartum haemorrhage** (n=135)	**Asthma** (n=352)	**Diabetic ketoacidosis** (n=252)	**Paediatric diarrhoea** (n=166)	**Injury** (n=1,627)	**Pneumonia** (n=761)	**Postpartum haemorrhage** (n=70)
Demographics												
Age group												
<5 years	8 (2.0)	2 (1.0)	182 (100.0)	46 (2.0)	32 (3.6)	0 (0.0)	26 (7.4)	0 (0.0)	166 (100.0)	183 (11.2)	143 (18.8)	0 (0.0)
5-<18 years	38 (9.4)	8 (4.0)	0 (0.0)	265 (11.6)	64 (7.2)	33 (24.4)	184 (52.3)	53 (21.0)	0 (0.0)	524 (32.2)	187 (24.6)	1 (1.4)
18-<60 years	255 (62.8)	126 (63.3)	0 (0.0)	1,700 (74.2)	555 (62.3)	102 (75.6)	41 (11.6)	80 (31.7)	0 (0.0)	723 (44.4)	305 (40.1)	69 (98.6)
≥ 60 years	105 (25.9)	63 (31.7)	0 (0.0)	281 (12.3)	240 (26.9)	0 (0.0)	101 (28.7)	119 (47.2)	0 (0.0)	197 (12.1)	125 (16.4)	0 (0.0)
Sex (female)	199 (49.0)	96 (48.2)	74 (40.7)	673 (29.4)	459 (51.5)	135 (100.0)	176 (50.0)	132 (52.4)	79 (47.6)	677 (41.6)	348 (45.7)	70 (100.0)
Facility disposition												
Discharged home	376 (92.6)	157 (78.9)	161 (88.5)	2176 (94.9)	683 (76.6)	130 (96.3)	323 (91.8)	213 (84.5)	150 (90.4)	1546 (95)	636 (83.6)	68 (97.1)
Left before completing care	4 (1.0)	4 (2.0)	3 (1.6)	17 (0.7)	15 (1.7)	1 (0.7)	6 (1.7)	11 (4.4)	7 (4.2)	17 (1.0)	23 (3.0)	0 (0.0)
Transferred to another facility	4 (1.0)	9 (4.5)	7 (3.8)	42 (1.8)	26 (2.9)	1 (0.7)	8 (2.3)	13 (5.2)	5 (3.0)	21 (1.3)	28 (3.7)	2 (2.9)
Death	22 (5.4)	29 (14.6)	11 (6)	57 (2.5)	167 (18.7)	3 (2.2)	15 (4.3)	15 (6.0)	4 (2.4)	43 (2.6)	74 (9.7)	0 (0.0)
